# A New Penetration Depth Method Using Proctor Compaction Test for Determining the Optimal Starting Time of Hardening Topping in Concrete Flooring

**DOI:** 10.3390/ma18215045

**Published:** 2025-11-05

**Authors:** Agnieszka Michalik, Jacek Zychowicz

**Affiliations:** 1 Building Structures, Geotechnics and Concrete Department, Building Research Institute, Filtrowa 1 Str., 00-611 Warsaw, Poland; 2 Faculty of Civil Engineering and Geodesy, Military University of Technology, gen. Sylwestra Kaliskiego 2 Str., 00-908 Warsaw, Poland; jacek.zychowicz@wat.edu.pl

**Keywords:** concrete floor, surface-hardened flooring, floating, dry shake topping, Proctor Compaction Test Apparatus, concrete mix setting rate, delamination, young concrete, early concrete strength

## Abstract

This article presents a development and validation of a method to determine the starting time for hardening concrete flooring mechanically floated using the Dry Shake Topping technique. Until now, an informal method based on shoeprint penetration depth of 3–4 mm into the hardening concrete floor has been used in practice, but it is prone to significant errors. The probe time method described in the literature also has multiple limitations and drawbacks. Currently, there is no scientifically verified method for accurately determining the setting time of concrete mix and its early compressive strength. This gap poses a research problem because incorrect early timing of topping floating leads to further defects in concrete flooring. Through various laboratory, pilot, and technical-scale tests, a new method was developed. According to this method, floating should begin when the penetration depth of the Proctor Compaction Test Apparatus in the concrete mix reaches 4–7 mm. This penetration depth corresponds to the point at which the hardening concrete mix achieves sufficient strength to support the floating equipment while remaining plastic enough to ensure a strong bond between the topping and concrete layers. The article presents correlations between the Proctor Compaction Test results and the early strength of young concrete. It also explains practical on-site application of the method, providing immediate results without the need for interpolation. This method can be applied to any concrete mix intended for use in concrete flooring.

## 1. Introduction

Flooring is the top layer of the floor and its outermost finishing coat. Concrete flooring is often used as a floor structure in industrial facilities, warehouses, commercial buildings, and garages [1,2,3,4,5]. Standard concrete floorings wear quickly due to frequent use and external loads, which results in surface degradation and excessive dusting. Various treatments, including but not limited to modifying the concrete’s composition by adding high-quality fibres and aggregates, are used to enhance the strength characteristics of floors and improve their abrasion resistance [6,7,8,9,10,11,12,13]. Surface treatment with dedicated materials composed of cement, sand, and admixtures or additives which create a hard and robust coating resistant to wear, impact, and mechanical damage is the most effective solution [14,15,16,17,18,19,20,21].

Dry Shake Topping (DST), which involves hardening the flooring surface with dry, cementitious toppings, is one of the most-used methods for finishing concrete flooring. Concrete industrial floorings with dry mineral toppings are most common in industrial buildings [22]. The DST consists of high-performance cements (class 52.5), which ensure the strength and durability of the layers, along with abrasion-resistant aggregates. Incorporating the topping at the wrong time can result in delamination or poor abrasion properties of the floor. Calcium aluminate cement (CAC) and Portland cement are used in DST floors [23]; these cements have increased resistance to abrasion, and the timing of installation is crucial for the quality of the floor. The final quality of industrial floorings with dry mineral toppings depends largely on the quantity of mineral topping used during the production process. This directly influences the floor’s abrasion resistance, which is crucial for the actual flooring’s durability and service performance. Additionally, the bond between the mineral topping layer and the concrete substrate is another critical factor. In brief, the execution of such floorings involves the following steps: concrete mix spreading, compacting with a vibrating float, smoothing the concrete mix and layer of water spontaneously released from the concrete (if applicable), waiting for the concrete mix setting to start, and spreading and floating the hardening topping [24,25].

Using the DST surface hardening method to finish the flooring provides unique characteristics by hardening the surface, reducing the risk of abrasion and mechanical damage, and creating a smooth and shiny top surface with a homogeneous colour [15,21,23,26]. However, it is challenging to fabricate surface-hardened floors. The critical parameter is to determine the starting time of the spreading and floating of the cementitious topping on the concrete mix surface. The time depends on several factors, including the concrete mix composition, consistency, gain rate of the concrete mix strength, ambient temperature, and humidity [27,28,29,30,31,32,33,34]. If the initiation time of floating the hardening topping is incorrectly determined, the procedure can start too soon or too late. Such timing errors can result in future defects in concrete flooring, including cracking of the hardened layer, delamination, colour differences, and non-uniform characteristics on the flooring surface, all of which can significantly affect the floor’s performance and durability [21,35,36]. Concrete flooring is susceptible to shrinkage and must be properly cured to prevent cracking and development of desired mechanical properties [22,23,37].

At present, no universally accepted technical test methods are available to determine the optimal initiation time for applying hardening topping during on-site floating and subsequent surface finishing using the DST method. The flooring contractors check the time using a technique that measures the shoeprint penetration depth, which should not exceed 3–5 mm upon stepping onto the concrete mix. Identifying the right moment depends on the flooring contractor’s experience as well as their weight or shoe size. However, this method is imprecise and may lead to execution difficulties, potentially resulting in improper floating of the hardening topping and subsequent flooring defects.

A literature review revealed publications on the test method for determining the initiation time of floating hardening topping [8,23,35,38,39,40]. A Dutch document, NEN 2743:2003 [38], describes the probe time method. The test shall be performed indoors at a temperature of 20 °C ± 2 °C. The method involves the following parameters: pouring the concrete mix into a ten-litre container using a probe with a right hammer, the total length of the probe should be 480 mm, the hammer’s weight should be 400 g, the diameter of the hammer base should be 200 mm, and the hammer drop height should be 250 mm. This method is used to determine the time after which the penetration depth of the twenty-five hammer drops at a single point in the concrete mix reaches 35 mm. The time refers to the moment when the floating of the hardening topping on the concrete flooring should begin. The test should be repeated three times for one concrete container. The result should be quoted as the mean of three measurements, with an accuracy of up to 1 mm. It also requires the repetition of the test every thirty minutes, or more often, and stopping the measurement when the penetration depth is less than 20 mm. The results should be presented in a graphic form as a diagram of the probe penetration depth (mm) versus time (h). The right initiation time of floating is then interpolated from the diagram.

The analysed Dutch method has some limitations: one should have the proper probe described in the document, the test is carried out under laboratory conditions, and the concrete mix is tested in plastic containers. All the aforementioned parameters do not adequately represent the actual performance of concrete flooring in situ. There are some doubts concerning the fact that the tests are performed under different conditions of concrete flooring setting (in a laboratory). In practice, floors are typically made at temperatures ranging from +5 °C to +30 °C, which may lead to substantial discrepancies between laboratory results and actual field performance.

Thus, the following scientific and research problem is addressed in this article: there is currently no universal testing method using measurement equipment to determine the optimal starting/initiation time for concrete flooring hardening using the Dry Shake Topping (DST) mechanical floating method. In practice, the concrete setting rate is determined by the person making the flooring, who checks the shoeprint penetration depth to ca. 3–5 mm. The concrete mix should be sufficiently set and strong to be stepped onto with the hardening topping floating equipment. On the other hand, it must not be fully set (hardened) as it would prevent the successful floating of the hardening topping. This is the only way to ensure adequate blending of the hardening layer with the fresh concrete. It affects the flooring’s final strength, durability, and visual characteristics and eliminates the risk of cracking and delamination of the hardening flooring layer. Although the literature describes the method for determining probe time [38], these approaches are not widely used due to equipment limitations, the method’s performance conditions, and the complexity of result interpretation. Therefore, a simple and rapid method for assessing the setting rate of fresh concrete to initiating the floating of the hardening topping is essential for the effective mechanical hardening of concrete floors using the DST method. Such a method should be universally applicable for any concrete formula.

Moreover, it is essential to determine the concrete’s actual compressive strength upon starting the hardening topping floating. New flooring concrete shall be strong enough to be stepped onto with a topping floating machine, yet sufficiently fresh to ensure effective and durable binding of the topping with the concrete. The compressive strength is determined based on the Proctor Compaction Test Apparatus drop depth, which complements the developed method.

The aim of the research is to define the simple and rapid method for determining the initiation time of concrete flooring floating using the Proctor Compaction Test Apparatus. The method builds upon existing practices by utilising a well-known, manual, and portable apparatus. It can be used in situ during the construction of mechanically floated floorings, which is the innovative aspect of the study. Using the Proctor Compaction Test Apparatus with the proposed method eliminates the need for low-precision shoeprint penetration testing, regardless of the concrete mix used for flooring applications. In practice, this will result in fewer incorrectly hardened floorings, where the topping floating was performed too soon or too late, leading to numerous surface defects in concrete floorings, such as microcracking or delamination of the layer. The developed method enables the use of the Proctor Compaction Test Apparatus, allowing for measurements on real floorings under any temperature conditions and providing quick and straightforward reading of the results, with no need for correlation.

The article presents the test results on the correlation between penetration depth measured with the Proctor Compaction Test Apparatus and the actual compressive strength of fresh concrete. The tests contributed to determine the strength level of the concrete mix at the initiation of the trowelling process for the hardening topping on the concrete surface. This testing approach represents a novel aspect, as no similar results were found in the reviewed literature.

## 2. Materials and Methods

### 2.1. Materials

**Figure 1:**
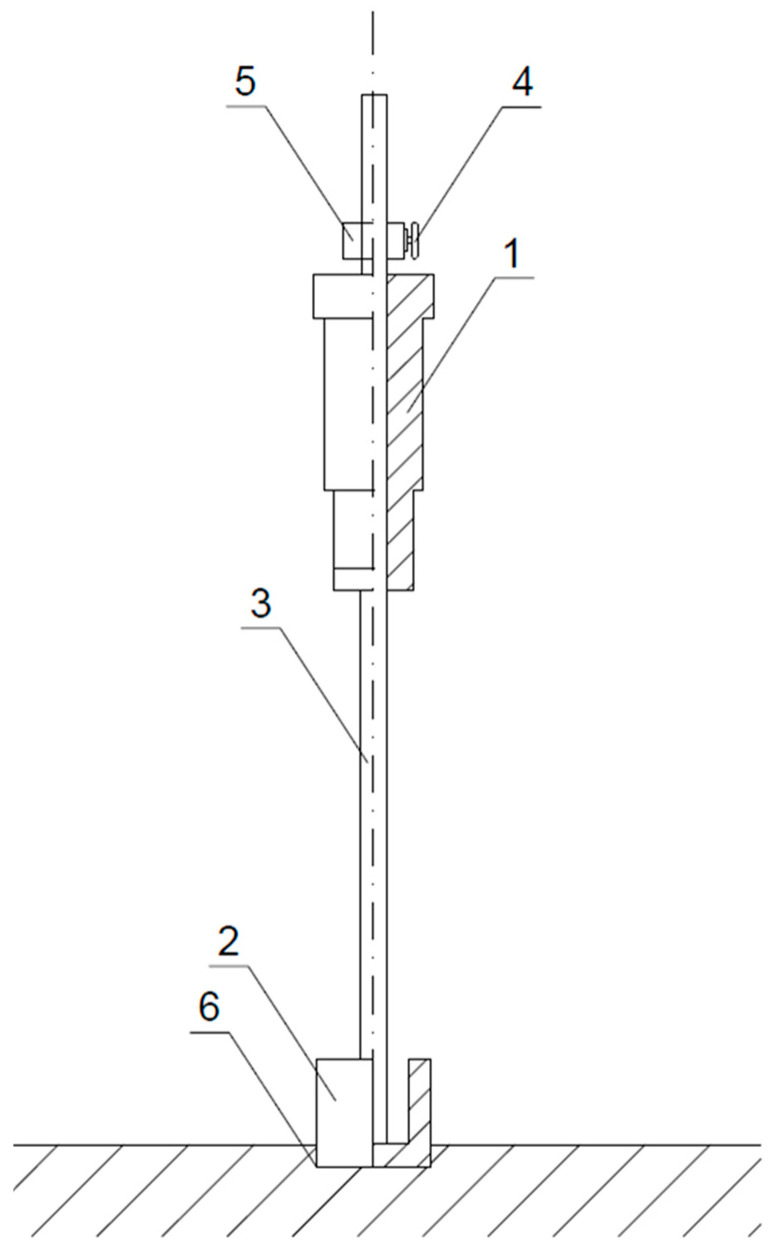
Proctor Compaction Test Apparatus: 1—rammer (hammer), 2—mould, 3—guiding bar, 4—screw, 5—drop height limiter, 6—concrete mix penetration depth.

To determine the initiating time of hardening topping floating, the authors sought a known construction sector device to eliminate the need to create a new piece of equipment. Since the device’s surface needed to be adequate to measure the setting rate of fresh concrete and its impact force should ensure penetration in the setting mix, two commercial Proctor Compaction Test Apparatuses, widely used in construction to determine soil compaction rate according to research standards [41,42], were selected. The technical parameters of the equipment are summarised in Table 1, and their design is shown in Figure 1. Both devices operate with the same compaction energies. Figure 1 shows a diagram of the Proctor Compaction Test Apparatus used in the referenced method to determine the commencement time of concrete flooring floating.

Table 2, Table 3 and Table 4 summarise the flooring concrete compositions used in the tests to determine the commencement time of the concrete flooring floating. For floorings, it is recommended to use cement ash with additives, such as fly ash, slag, and limestone, whose hydration heat is lower and the strength development is moderate. CEM I is not recommended due to high hydration heat and the risk of concrete flooring cracking. In the first stage of this study, a mixture of CEMI 42.5R NA with CEM III A 42.5N LH HSR NA was used (which is similar to the CEM II/B-S properties). In the next stage, CEM II/A-M (S-LL) 52.5N and fly ash were used as concrete additives. Fly ash is generally not recommended for DST-hardened floorings due to surface finishing issues (discolouration, ash “flowing out”), flaking of the surface layer, the risk of the additives’ segregation/separation, and variable quality of the raw material [43,44,45]. Still, the additive tends to be used in such floorings due to reduced hydration heat, improved workability, and environmental aspects. River sand (0–2) is used as aggregate, along with cobble gravel (2–16) as coarse aggregate, commonly added to floorings. Their mixing ratio was typical for concrete floorings. The concrete compositions were modified with chemical admixtures (fluidising and aerated) and with variable amounts of water to obtain variable characteristics of the concrete mix, varying in consistency and aeration. A superplasticiser based on lignosulphonates, common for flooring concrete, was used as an admixture.

### 2.2. Methods

**Figure 2:**
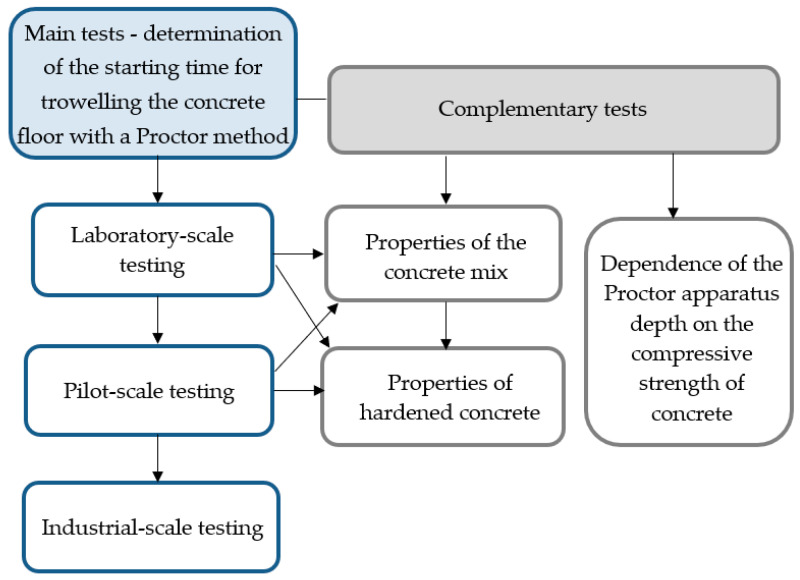
Research programme.

The general research programme is presented in Figure 2.

Determination of the starting time of cementitious topping floating with a Proctor Compaction Test Apparatus is the primary method presented in this article. Table 1 summarises the parameters of the applied apparatuses that vary slightly for the hammer weight and base diameter. However, their compaction energy is the same, and hence, they can be used interchangeably. The method was prevalidated in the laboratory and then under pilot and industrial conditions. The mix was poured into 50 × 50 × 17 cm moulds (the mould height was the same as that of real floorings), compacted with a vibrator and smoothed. Shoeprint measurements were then carried out at regular time intervals (up to 30 min); the footprint impression was of a male weighing 90 kg and wearing a shoe size 42. It was followed by measurements of the Proctor hammer drop depth.

The method is characterised by the concrete mix hardening being checked every 30 min or more often using the Proctor Compaction Test Apparatus. The first test was performed one hour after the concrete mix was spread. The apparatus was placed on the tested concrete mix surface, and the hammer was lifted to the drop height limiter and released. The hammer was dropped onto the mould bottom and formed a dent in the topcoat of the concrete mix. Subsequently, the hammer penetration depth was measured at the lowest point using a ruler with an accuracy of up to 1 mm. Next, two test cycles were performed at two measurement points located at least 50 mm apart, and the mean of the three penetration depth measurements was calculated with an accuracy of up to 0.1 mm. The mean of the three measurements that fell within the range of 4–7 mm was the starting time of the hardening topping floating. The starting time of floating determines the concrete mix hardening. The 4–7 mm value of the Proctor Compaction Test Apparatus penetration depth was correlated on laboratory, pilot, and technical scales with a shoeprint penetration depth based on several measurements for various concrete mixes performed in different environmental conditions.

The relationship between the Proctor Compaction Test Apparatus penetration depth and the actual development of fresh concrete compressive strength were also evaluated. The test was performed to estimate the concrete strength during hardening, allowing topping floating works to commence. This is the innovative aspect presented in the article. Concrete mixes described in Table 2 as Concrete I-IV were spread in several steel moulds, sized 150 *×* 150 *×* 150 mm. The moulds were dismountable to facilitate removing fresh concrete with no damage. Other tests carried out on the specimen in the mould included the Proctor Compaction Test Apparatus, penetration depth measurement, and compressive strength testing.

Moreover, the following tests were performed for concrete mixes and hardened concrete:

- Slump test (for consistency) according to EN 12350-2:2019 [46];
- Air content in the concrete mix according to EN 12350-7:2019 [47];
- Concrete mix density according to EN 12350-6:2019 [48];
- Flexural strength for three specimens sized 100 × 100 × 500 mm according to EN 12390-5:2019 [49];
- Compressive strength for three specimens sized 150 *×* 150 *×* 150 mm according to EN 12390-3:2019 [50].

The performed methods were selected to fully characterise the tested flooring concrete, including the consistency class, air content value, flexural strength, and compressive strength.

## 3. Results

### 3.1. Laboratory-Scale Testing

The possibility of developing a method to determine the measurable initiating time for floating concrete flooring was analysed; the available commercial technical equipment was utilised for this purpose. The moment of starting the floating is determined by a floating person’s shoeprint. It should range from 3 to 5 mm in depth, but it depends on the person’s weight and shoe size. Alternatively, the initiation time for floating can be determined based on experience. Changes in the mix composition, cementitious additives, and many other factors contribute to floating too soon or too late, thereby increasing the risk of flooring surface defects. The study included attempts to correlate the shoeprint with the penetration depth of the Proctor Compaction Test Apparatus (typically used for soils) according to PN-B-04481:1988 [41] or EN 13286-2:2010 [42]. The two types of apparatuses presented in Table 1 were used.

**Figure 3:**
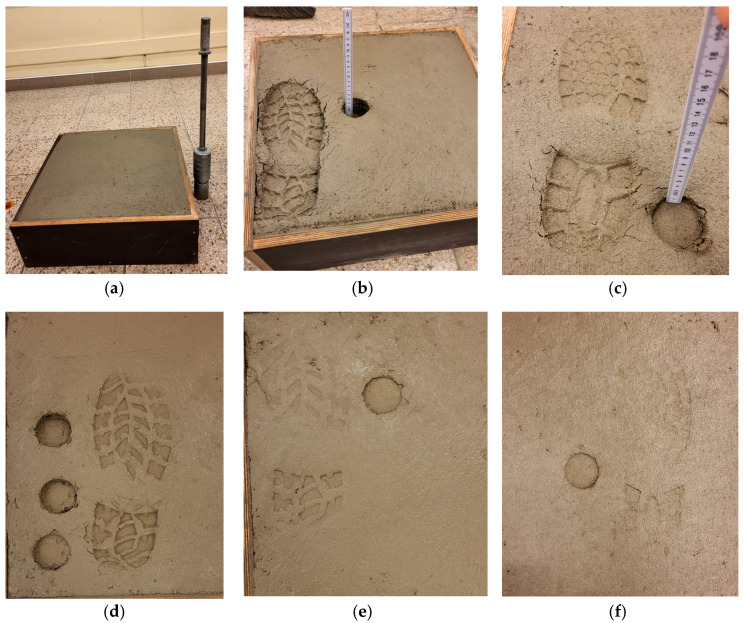
Laboratory-scale tests of the shoeprint and Proctor Compaction Test Apparatus penetration depth, (**a**) test rig, and (**b**–**f**) consecutive testing stages after 1 h (**b**), 1.5 h (**c**), 2 h (**d**), 2.5 h (**e**), 3 h (**f**).

Concrete mixes were prepared with compositions typical of flooring concrete, according to the composition summarised in Table 2 and Table 3. Figure 3 shows the images of the shoeprint and Proctor Compaction Test Apparatus’ penetration depth performed in the laboratory-scale tests.

Laboratory tests were repeated four times using different compositions of the concrete mix in each test, reflecting common flooring concrete formulas according to Table 2 and Table 3. The environmental conditions in the testing laboratory were controlled with a thermo-hygrometer. The air temperature ranged from 20 to 23 °C, and the relative humidity ranged from 40 to 50%. The penetration depths of the shoeprint and the Proctor apparatus were measured. The shoeprint penetration depth of 3–5 mm was the criterion for starting the topping floating. This is a standard value used in practice. The test involved identifying the Proctor hammer penetration depth that corresponds to the shoeprint penetration depth of 3–5 mm. The averaged results are shown in Table 5.

The term “starting time” refers to the degree of concrete mix setting at which work on troweling the hardening topping onto the surface of a concrete floor made using the Dry Shake Topping method can begin. The starting time is determined when the average of three Proctor depth measurements is within the range of 4–7 mm.

**Figure 4:**
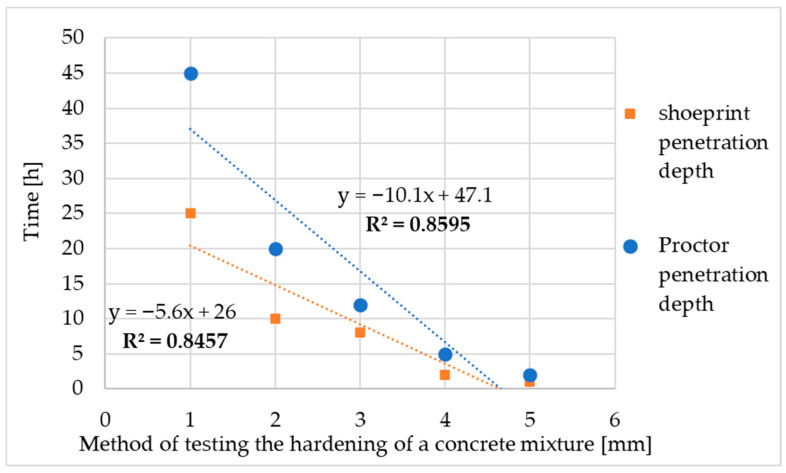
The correlation between shoeprint penetration depth and Proctor penetration depth.

Laboratory tests revealed a correlation between the shoeprint and the Proctor apparatus penetration depths. The Proctor apparatus penetrates a few millimetres deeper than a shoe in the same time. Laboratory validations served as the basis for testing the method on a pilot scale. The correlation results presented in Table 5 are graphed along with the trend line equation and the coefficient of determination R^2^. The coefficient of determination R^2^ is approximately 0.9 for both the shoeprint depth and Proctor methods, indicating a strong correlation between the time results and the concrete set degree testing. This confirms the validity of the developed Proctor method. The correlation between shoeprint penetration depth and Proctor penetration depth is shown in Figure 4.

**Figure 5:**
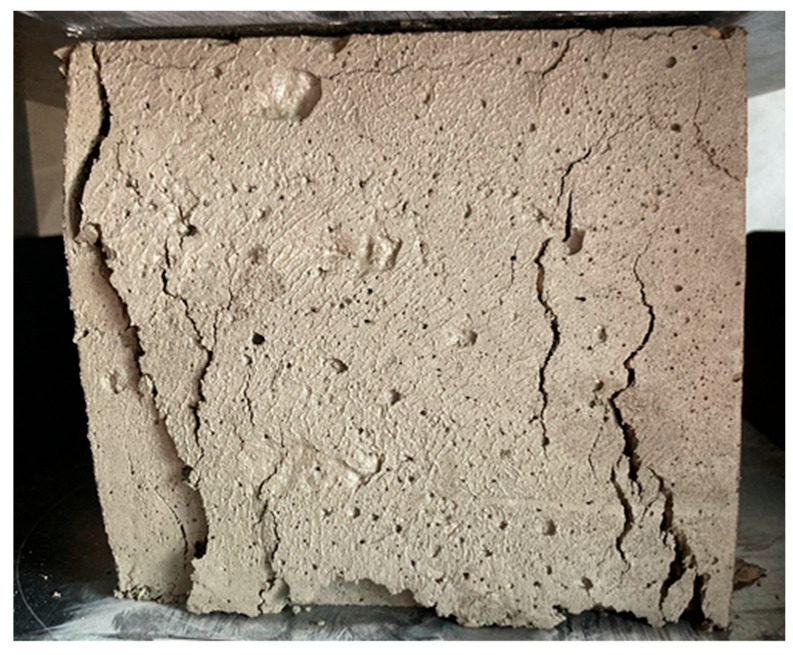
Image of damaged fresh concrete 3 h 15 min after producing the mix.

The results of tests on the relationship between the Proctor Compaction Test Apparatus and the actual compressive strength development in fresh concrete are summarised in Table 6. Strength tests were performed simultaneously with the Proctor Compaction Test Apparatus penetration. The image of damaged fresh concrete 3 h 15 min after producing the mix is shown in Figure 5.

The test results summarised in Table 5 reveal that when the penetration depth of the Proctor Compaction Test Apparatus reaches ca. 5 mm, the concrete’s compressive strength is ca. 0.2 MPa. This is the right moment to start floating the hardening topping. Moreover, the fresh concrete compressive strength, ranging from 0.2 to 0.3 MPa, was found to be adequate for topping floating with the DST method. When the compressive strength value exceeds 0.3 MPa, the concrete is too set to execute the works correctly. This article presents test results for Concrete I, while the test was conducted for Concrete I-IV in the temperature range of 5–30 °C and relative humidity of 40–80%, with repeatable results. These preliminary results provide a basis for further investigation of this relationship on a larger scale, which will be the subject of the next article.

### 3.2. Pilot-Scale Testing

**Figure 6:**
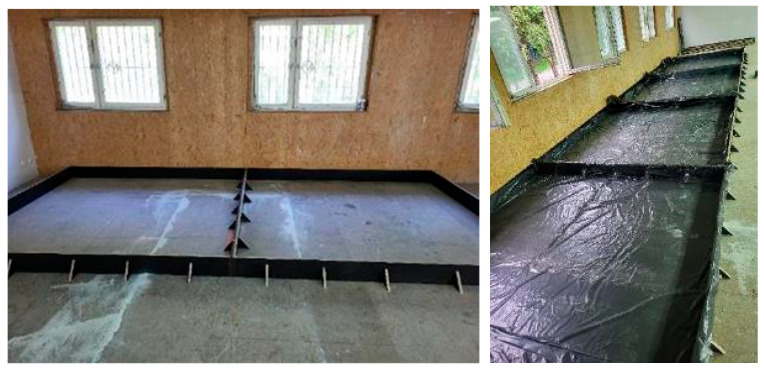
Test fields were prepared for laying the flooring on a pilot scale.

In order to make industrial floorings on a pilot (semi-technical) scale, test fields for floorings were prepared. The dimensions of the test fields must be sufficiently large to float the hardening topping correctly using floats and discs. In the first stage, four test fields were used, each of approximately 250 cm × 250 cm × 17 cm in size. The test fields were laid with foil to allow for free sliding of the flooring during shrinkage and to prevent water from draining through the substrate. The test fields are shown in Figure 6.

**Figure 7:**
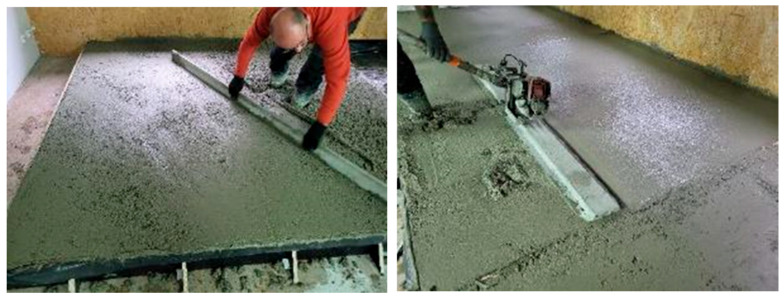
Flooring smoothing and compacting.

A concrete mix whose composition is specified in Table 2 (Concrete I and II) was used for the first pilot-scale tests; it was supplied from a ready-mixed concrete production plant. The mixture in each test field was smoothed and compacted by using a power screed. The compaction process is illustrated in Figure 7.

**Figure 8:**
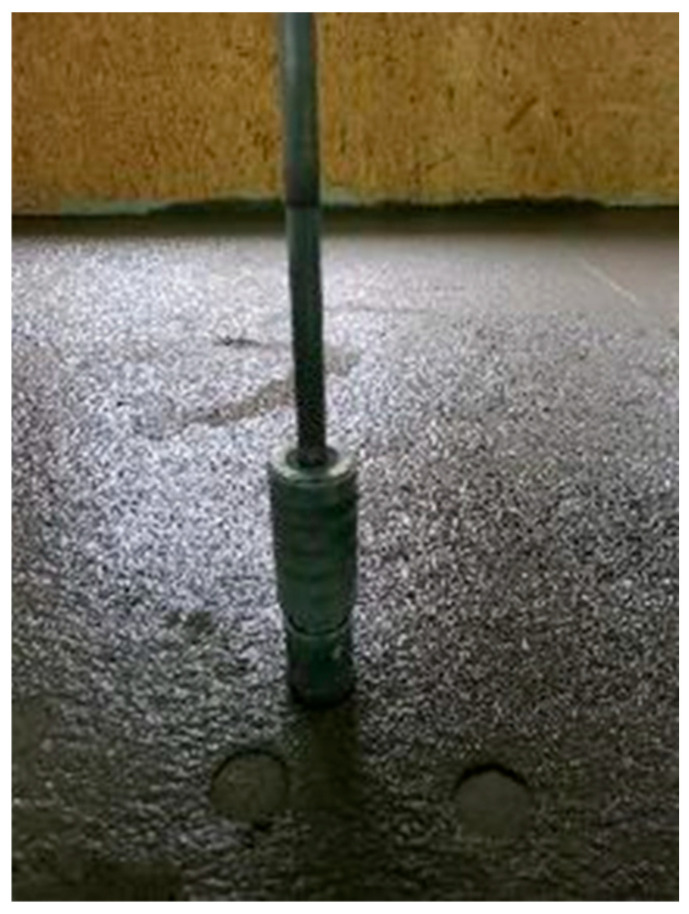
Proctor method testing—pilot scale.

**Figure 9:**
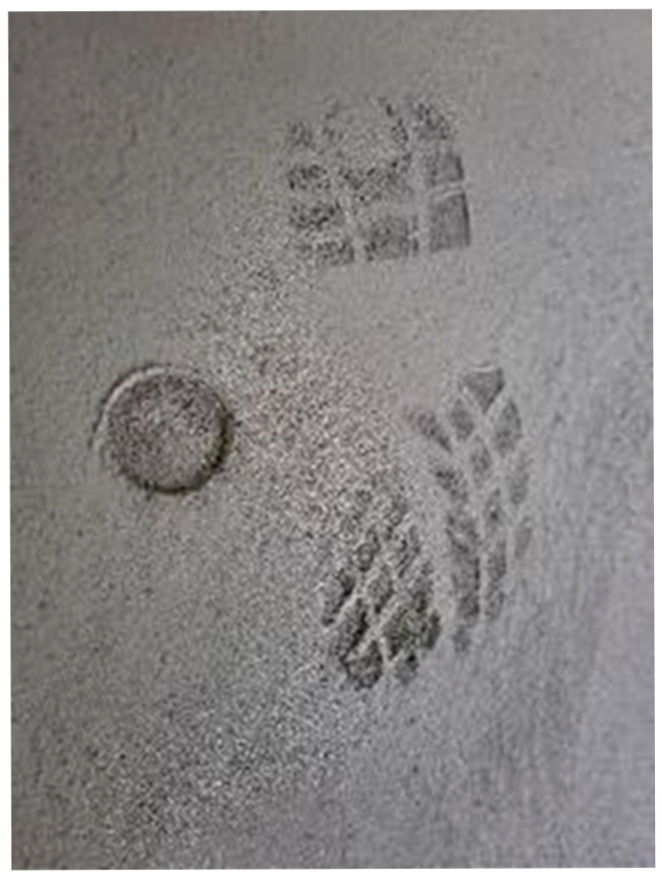
Correlated starting time for floating the hardening topping was determined using a shoeprint and Proctor apparatus on a pilot scale.

Then, about one hour after spreading and compacting the mix, the hardness measurement of the concrete mix began. The mix setting was checked simultaneously by an experienced employee from a company that manufactures mechanically hardened concrete floors (with a shoeprint—stepping onto the flooring) and by a laboratory staff member using a Proctor Compaction Test Apparatus at a nearby point. Initially, the measurements were performed every 30 min, and then every 15–20 min after the mix started to set. The view of the pilot-scale measurements is shown in Figure 8 and Figure 9.

In the subsequent stages, measurements were performed for other floorings of different compositions, as listed in Table 3 and Table 4 (Concrete III, IV, V, and VI). Comparative pilot-scale tests revealed that the optimal starting time for flooring hardening, that is, floating the hardening topping with discs, is when the penetration depth in the mix is approximately 3–5 mm for the shoeprint and 4–7 mm for the Proctor Compaction Test Apparatus. Two complete measurements were performed on the pilot scale for each mixture, making a total of twelve measurements.

The Proctor device has a diameter of 50 mm, while the aggregate grain size is a maximum of 16 mm, which does not affect the measurement. In properly prepared concrete, the aggregate is embedded in the concrete mix and does not rise to the surface. The Proctor rammer’s drop is single, and during the initial setting phase of the mix, when the rammer strikes the aggregate, the grain sinks into the mix. However, in the final setting phase, the rammer only penetrates a few mm and does not contact the coarse aggregate. Furthermore, measurements are taken at three adjacent locations, and the final result is the average of the three measurements. Tests showed that the result was repeatable in each of the three measurements, indicating that the aggregate does not affect the consistency of the device’s readings.

It is worth noting that at each stage of pilot-scale testing, indoor environmental conditions were recorded using a thermo-hygrometer. The floorings were made at temperatures ranging from 5 to 30 °C and relative humidity from 40 to 85%. Table 7 summarises the test results on the characteristics of concrete mixes and hardened concrete.

Various concrete mix compositions (Table 2, Table 3 and Table 4) were designed and tested on a pilot scale to correlate the shoeprint penetration with the Proctor apparatus penetration method for mixes with different characteristics. As shown in Table 6, the tested mixes varied in their consistency classes and air content, ranging from 0.5% to 10%. The compressive strength values of the tested concrete specimens also ranged from low (16 MPa) to high (42 MPa). Regardless of the concrete composition and environmental conditions, the concrete mix intended for flooring finished with the DST method shall be set sufficiently after spreading so that it can be stepped onto with the topping floating equipment, but not so much that it prevents effective blending of the hardening layer with the concrete. Concrete composition and ambient (environmental) conditions affect the moment of starting the floating. It can range from a few to several hours after spreading the mix. Testing with the Proctor Compaction Test Apparatus helped in the precise determination of the floating starting time for all analysed and diversified concrete mixes. This confirms the universal nature of the methods and develops the previously described testing methods.

### 3.3. Industrial-Scale Testing

Successive tests were conducted on a technical scale during the actual floor making, as illustrated in the example. A total of 482 m^3^ was delivered from the ready-mix concrete production plant to the site where the concrete flooring was to be made. The concrete flooring area was 3210 m^2^, and it was approximately 0.15 m thick. It was made for a door manufacturing company. The concrete mix composition and properties were adequate for industrial flooring: consistency class S3, compressive strength class C20/25, exposure class XC2, and the maximum aggregate grain size of 16 mm, CEM II BS 42.5 N cement and gravel aggregates. The temperature in the room where the flooring was made was 19.6 °C, and the relative humidity was 44.2% when the test was performed. The concrete mix was spread and compacted with a power screed. An hour later, the tests started. The first test with a Proctor Compaction Test Apparatus (shown in Figure 1) was conducted one hour after spreading the concrete mix. Then, two consecutive test cycles were carried out at other measurement spots, spaced at least 150 mm apart from one another and from the previous measurement spot. The mean of the three measurements was determined with an accuracy of up to 0.1 mm. The measurement results are summarised in Table 8.

The technical-scale tests with the Proctor apparatus were repeated a few times in server rooms and industrial facilities where DST floorings were made, in cooperation with a leading concrete manufacturer. The tests in this study were performed interchangeably by at least six people, and the results were reproducible; no operator-dependent dependence of penetration depth was observed.

According to the laboratory- and pilot-scale validation tests, the adequate starting time for hardening topping floating is when the mean of three measurements with the Proctor Compaction Test Apparatus ranges between 4 and 7 mm. In the presented case, on an industrial scale, the mean measurement after three hours was 4.3 mm. This moment was set as the starting timepoint for the floating of the hardening topping with the discs. The surface was then smoothed using floor-smoothing paddles. The embedded topping formed a consolidated, hardened layer on the concrete flooring, confirming the efficacy of the Proctor Compaction Test Apparatus in determining the starting time of topping floating.

## 4. Discussion

Concrete floorings with surfaces hardened with the DST method are the most common finishing type of flooring surface due to their smoothness, high strength, abrasion resistance, and surface tightness. Determining the optimal starting time for surface hardening is crucial to achieving the required flooring performance parameters. Therefore, there is a need for a universal, practical, and repeatable in situ testing method that utilises commercially available equipment.

In current practice, the shoeprint method is routinely used to assess the setting of the concrete mix. However, this method is imprecise and prone to numerous errors, as it depends on various factors such as the tester’s body weight and shoe size. For example, the shoeprint penetration depth differs between individuals weighing 80 kg and 100 kg or wearing shoes with different sizes. Such variability can lead to inaccurate assessments resulting in flooring defects or subsequent complaints.

The literature describes a standardised procedure for determining the optimal starting time for concrete floor hardening in the Dutch standard NEN 2743:2003 [38], which outlines the probe time method. However, this method is complex and has several limitations. It requires laboratory conditions (20 ± 2 °C) and testing of the concrete mix in ten-litre containers. Additionally, the probe used in this method has a diameter of 200 mm (probe weight: 400 g, drop height: 250 mm, length: 480 mm), which is insufficient for concrete mixes containing aggregates with a maximum size of 16 or 32 mm. There is also the risk when the probe hits the aggregate grain, which can affect measurement accuracy. In the probe time method [30], the time is determined after which the depth of the twenty-five hammer drops in one measurement point amounts to 35 mm. The test shall be repeated three times per container with concrete, with the result quoted as a mean of three measurements with an accuracy of up to 1 mm. The test should be repeated every thirty minutes, requiring a high number of containers with concrete mix. The measurement can be stopped when the penetration depth is less than 20 mm. The results should be presented in a graphic form, as a diagram of the penetration depth (mm) versus time (h). The end time is extrapolated from the diagram. In practice, a quick method for investigating the setting rate of concrete mix is expected so as not to miss the moment when surface hardening with topping should start. These factors make this method difficult to apply in practice, leading to its infrequent use.

To address these issues, a new approach was developed based on numerous validation tests conducted under laboratory conditions, followed by pilot- and industrial-scale trials. This method determines the setting rate of concrete mixes to establish the appropriate time for commencing surface hardening with a topping. It employs the Proctor Compaction Test Apparatus, a device commonly used in construction for soil compaction.

This proposed method offers several advantages, such as a diameter of probe suitable for testing concrete mixes (50 mm in diameter, hammer weight: 4.5 kg, drop height: 457 mm, length: 610 mm), and it uses a mobile construction device, allowing for easy on-site use. Results are obtained immediately, and the method does not require complex laboratory procedures. The depth obtained in the Proctor method is identified when one hammer drop is 4–7 mm in depth. The result is quoted as the mean of three measurement points, positioned at least 50 mm apart, with an accuracy of up to 1 mm. Initially, the test is repeated every thirty minutes. When the mix starts curing, it is repeated every 15–20 min. The results are obtained in situ, which is a significant improvement over the Dutch method. Moreover, the Proctor method can be applied to different concrete mixture compositions, regardless of ingredients or concrete mix characteristics, and under various environmental conditions (e.g., different temperatures and humidity levels). Additionally, this research involves the determination of the early strength of fresh, setting concrete when the hardening topping is floated, which was not previously addressed in the literature. The test results on the Proctor apparatus penetration depth and simultaneous compressive strength tests reveal that floating of the hardening mix with floats and discs on the flooring surface with the DST method can start when the concrete’s compressive strength falls within the 0.2–0.3 MPa range.

The obtained results indicated that the procedure of spreading the hardening topping on the concrete mix surface and its subsequent floating can begin when the mean penetration depth of three Proctor apparatus measurements falls between 4 and 7 mm.

This method provides a technical and reliable alternative to the traditional shoeprint approach, effectively eliminating the uncertainties associated with manual assessment.

The presented Proctor Compaction Test Apparatus penetration method has several advantages: it utilises known mobile equipment commonly used in construction, the results are instantly available, and the tests can cover various concrete mixes, allowing for performance under different environmental conditions. Moreover, the work determined the actual compressive strength at the moment of starting to trowel the hardening powder onto the floor surface, which is a novelty in this article.

The method is certainly limited by the use of dense reinforcement concrete. Furthermore, application limitations include errors in the concrete mix composition, such as excessively fluid consistency, mix segregation, or high fly ash content. Assuming the concrete mix has the appropriate composition and properties, the method is effective.

## 5. Conclusions

This study proposes a practical and reliable method for determining the optimal starting time for concrete flooring hardening using dry toppings and their floating, using the Proctor Compaction Test Apparatus. Unlike traditional shoeprint or probe methods, it provides accurate, repeatable, and on-site measurements without complex laboratory procedures. The method is applicable to various concrete mixes and environmental conditions.

1. Results indicate that the topping and floating process should begin when the mean Proctor penetration depth is 4–7 mm, ensuring proper surface quality and reducing the risk of defects. This approach offers a standardised and objective alternative for flooring practice.
2. The test results on the Proctor apparatus penetration depth and simultaneous compressive strength tests reveal that floating of the hardening mix with floats and discs on the flooring surface with the DST method can start when concrete’s compressive strength falls within the 0.2–0.3 MPa range.
3. The presented universal method for determining the starting time of hardening topping floating is used in real in situ conditions, at the temperature range between +5 °C and +30 °C and humidity conditions between 40% and 85%, when executing surface-hardened indoor and outdoor concrete floorings for all types of concrete mixes, regardless of the ingredients or concrete mix characteristics. These results are readily available in situ owing to the manual Proctor Compaction Test Apparatus, well known in construction.
4. In reference to well-known solutions, the method described in the article provides a simple and sufficiently accurate way to determine the starting time of concrete flooring hardening using dry toppings and their floating. The identified starting time of the finishing works testifies to the hardening of the concrete mix and the possibility of spreading the hardening topping on the concrete mix surface and its subsequent floating. Performing finishing works on a concrete mix with a specific hardness ensures a good flooring performance.
5. The presented Proctor method helps to technically eliminate the previously common way of checking concrete mix hardness by stepping onto the flooring and examining the shoeprint, which suffers from many uncertainties. It also develops the probe time method described in the literature, which had many limitations in practical application.
6. The method has the following limitations: the suitability of this method was tested at a temperature range from +5 °C to +30 °C and a relative humidity of 40–85%. The method is certainly limited by the use of densely reinforced concrete floors. Furthermore, application limitations include errors in the concrete mix composition, such as excessively fluid consistency, mix segregation, or high fly ash content. Assuming the concrete mix has the appropriate composition and properties, the method is effective. Weather conditions, such as rain, are also limitations, but this is also a limitation for other concrete methods.

## 6. Patents

Patent application title: Method for Determining the Start Time of Concrete Flooring Surface Hardening Works. Application No. P.452248. Invented by: Agnieszka Michalik and Jacek Zychowicz.

## Figures and Tables

**Table 1:** Proctor Compaction Test Apparatuses used in the method.

Apparatus Application Standard	PN-B-04481:1988 [41]	EN 13286-2:2010 [42]
Hammer type per standard	Method I, lightweight/small hammer	A
Hammer base diameter [mm]	50.8	50.0
Hammer weight [kg]	2.5	2.5
Hammer free fall height [mm]	320	305
Cylindrical mould diameter [mm]	100	100
Compaction energy [kJ/m^3^]	600	600

**Table 2:** Composition of the flooring concrete I and II.

Ingredient	Concrete I	Concrete II
	Content [kg/m^3^]	Content [kg/m^3^]
Portland cement CEM I 42.5 R NA	150	150
Blast furnace cement CEM III A 42.5N LH HSR NA	150	150
Tap water	150	180/170
Fine aggregate 0/2 (river sand)	7301	7301
Coarse aggregate 2/16 (cobble)	1130	1130
Superplasticiser	3.0	3.0

**Table 3:** Composition of the flooring concrete III and IV.

Ingredient	Concrete III	Concrete IV
	Content [kg/m^3^]	Content [kg/m^3^]
CEM II/B-S 32,5 R	300	300
Tap water	150	150
Fine aggregate 0/2 (river sand)	7301	7301
Coarse aggregate 2/16 (cobble)	1130	1130
Superplasticiser	3.0	3.0
Aerating (air-entraining) admixture	-	3.0

**Table 4:** Composition of the flooring concrete V and VI.

Ingredient	Concrete V	Concrete VI
	Content [kg/m^3^]	Content [kg/m^3^]
CEM II/A-M (S-LL) 52.5N	200	200
Silica fly ash	80	80
Tap water	168	190
Fine aggregate 0/2 (river sand)	750	750
Coarse aggregate 2/16 (cobble gravel)	1090	1090
Superplasticiser, fluidising admixture	1.5	5.5
Plasticiser, plasticising admixture	0.7	0.7
Aerating (air-entraining) admixture	3	3

**Table 5:** Results of laboratory tests.

Time After Spreading the Mix [h]	Shoeprint Penetration Depth [mm]	Proctor Hammer Penetration Depth [mm]
1	25 ± 2	45 ± 2
1.5	10 ± 2	20 ± 2
2	8 ± 2	12 ± 2
2.5	2 ± 2	5 ± 2
3	1 ± 2	2 ± 2

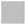
—the correlated starting time of floating is highlighted in grey.

**Table 6:** Correlation between Proctor apparatus penetration and compressive strength—test results.

Time After Spreading the Mix [h]	Proctor Hammer Penetration Depth [mm]	Compressive Strength [MPa]
1 h	50 ± 2	0
1 h 30 min	42 ± 2	0
2 h	17 ± 2	0
2 h 30 min	12 ± 2	0
3 h 15 min	5 ± 2	0.21
4 h	2 ± 2	0.29
5 h	1 ± 2	0.43

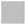
—the correlated time of starting the floating is highlighted in grey.

**Table 7:** Concrete testing results.

Concrete Type	Consistency—Slump Test [mm]/Consistency Class	Air Content in Concrete Mix [%]	Concrete Mix Density [kg/m^3^]	Flexural Strength After 28 Days [MPa]	Compressive Strength After 28 Days [MPa]
I	120/S3	4.0	2290	6.0	41.1
II	220/S4	2.8	2300	5.2	27.1
III	120/S3	4.0	2325	5.4	42.1
IV	160/S3	10.0	2290	3.9	18.7
V	210/S4	4.8	2260	3.5	17.5
VI	self-levelling mix	0.5	2315	2.9	16.0

**Table 8:** Industrial-scale test results.

Time After Spreading the Mix [h]	Proctor Compaction Test Apparatus Penetration Depth [mm]	
	Individual Measurements	Mean
1	45, 48, 47	46.7
1.5	23, 25, 20	22.7
2	12, 15, 14	13.7
2.5	8, 8, 10	8.7
3	4, 5, 4	4.3

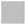
—the determined time of starting the floating with the Proctor method is highlighted in grey.

## Data Availability

The original contributions presented in this study are included in the article. Further inquiries can be directed to the corresponding author.

## Abbreviation

The following abbreviation are used in this manuscript:

DST	Dry Shake Topping
